# Role and response of primary healthcare services in community end-of-life care during COVID-19: Qualitative study and recommendations for primary palliative care delivery

**DOI:** 10.1177/02692163221140435

**Published:** 2023-02

**Authors:** Nicola Turner, Aysha Wahid, Phillip Oliver, Clare Gardiner, Helen Chapman, Dena Khan (PPI co-author), Kirsty Boyd, Jeremy Dale, Stephen Barclay, Catriona R Mayland, Sarah J Mitchell

**Affiliations:** 1 University of Sheffield, Sheffield, UK; 2 Sheffield Teaching Hospitals NHS Foundation Trust, Sheffield, UK; 3 Patient and Public Involvement Representative, University of Sheffield, UK; 4 University of Edinburgh, Edinburgh, UK; 5 University of Warwick, Warwickshire, UK; 6 University of Cambridge, Cambridge, UK

**Keywords:** Palliative care, terminal care, COVID-19, primary health care, general practice, primary care nursing, qualitative research

## Abstract

**Background:**

The need for end-of-life care in the community increased significantly during the COVID-19 pandemic. Primary care services, including general practitioners and community nurses, had a critical role in providing such care, rapidly changing their working practices to meet demand. Little is known about primary care responses to a major change in place of care towards the end of life, or the implications for future end-of-life care services.

**Aim:**

To gather general practitioner and community nurse perspectives on factors that facilitated community end-of-life care during the COVID-19 pandemic, and to use this to develop recommendations to improve future delivery of end-of-life care.

**Design:**

Qualitative interview study with thematic analysis, followed by refinement of themes and recommendations in consultation with an expert advisory group.

**Participants:**

General practitioners (*n* = 8) and community nurses (*n* = 17) working in primary care in the UK.

**Results:**

General practitioner and community nurse perspectives on factors critical to sustaining community end-of-life care were identified under three themes: (1) *partnership working* is key, (2) *care planning* for end-of-life needs improvement, and (3) importance of the *physical presence* of primary care professionals. Drawing on participants’ experiences and behaviour change theory, recommendations are proposed to improve end-of-life care in primary care.

**Conclusions:**

To sustain and embed positive change, an increased policy focus on primary care in end-of-life care is required. Targeted interventions developed during COVID-19, including online team meetings and education, new prescribing systems and unified guidance, could increase capacity and capability of the primary care workforce to deliver community end-of-life care.

**What is already known about the topic?**

- The COVID-19 pandemic was associated with at least 3 million excess deaths internationally, with a significant increase in the number of people dying at home, including in care homes.
- Attempts were made to rapidly implement changes in individual practice and primary care service delivery to provide community end-of-life care, but sometimes had unintended consequences.
- Increased use of virtual consultations by general practitioners led to community nurses reporting a sense of abandonment as they continued to deliver care in the home.

**What this paper adds?**

- Multi-professional, cross-boundary working between primary care and specialist palliative care services can enhance both the physical and psychological ability of professionals to engage in community end-of-life care when there is a rapid increase in demand.
- Time and resource in primary care for communication and end-of-life care planning with patients is required to support motivated staff already seeking to create care planning opportunities and manage emotional demands.
- End-of-life care in the community requires the physical presence of all members of the primary healthcare team in both the delivery of frontline, face-to-face care and in healthcare system leadership.

**Implications for practice, theory or policy**

- Recommendations for future practice and policy include maintaining cross-boundary team meetings and online education sessions between primary care and specialist palliative care.
- Effective communication and end-of-life care planning with patients requires the allocation of time and resource in primary care, where most end-of-life care is delivered.
- Clear, consistent, and unified guidance is necessary for primary care professionals during times of increased demand for community end-of-life care, such as pandemics.

## Introduction

The COVID-19 pandemic was associated with at least 3 million excess deaths internationally, with many more deaths in community settings.^1^ Primary healthcare professionals, including general practitioners and community nurses, have a key role in the delivery of care to people who die at home.^2^ However, there was scarce evidence to inform primary care policy and practice in relation to the delivery and sustainability of community end-of-life care during the COVID-19 pandemic.^3^

The disruption to healthcare services resulting from the pandemic generated new and unexpected opportunities for cross-boundary working and innovation in community end-of-life care.^4,5^ Primary healthcare professionals had to adapt quickly to provide end-of-life care to increased numbers of patients.^6,7^ Very little research exists to understand the role of primary care in end-of-life care during the pandemic. A primary care survey (conducted across the United Kingdom by this team) found that service changes in response to infection control measures, such as increased virtual consultations, appeared to have some benefits for patient care, but there were also unintended consequences. Notably, community nurses reported a sense of abandonment and emotional distress while taking on more responsibility for face-to-face care in the home.^6^

Increased understanding about what works in the delivery of community end-of-life care at times of increased demand is vital, including from the perspective of primary care. The aims of this study were:

(1) to gather detailed insights from the perspectives of general practitioners and community nurses on factors that enabled the delivery of community end-of-life care during the COVID-19 pandemic, and
(2) to develop recommendations to improve primary care delivery of end-of-life care, including during pandemics and other times of increased need.

## Methods

### Study design

A descriptive, qualitative study using virtual semi-structured interviews to explore individual perspectives on the delivery of community end of life care during the COVID-19 pandemic. The study is reported in keeping with the Standards for Reporting Qualitative Research (SRQR).^8^

### Setting

This study was the second part of a mixed method investigation of the role and response of United Kingdom primary healthcare services in the delivery of end-of-life care during COVID-19. The first part of the study has been reported previously, and comprised a web-based, questionnaire survey completed by 559 general practitioners and community nurses who were recruited via local and national professional networks.^6^ Participants who expressed an interest in an interview after completing the survey were invited to take part in phase two.

### Recruitment

Of the 196 survey respondents contacted 127 did not reply, 15 were no longer available at the email address provided, 3 declined, and 51 responded positively. No data was collected on why eligible participants chose not to respond. Up to three attempts were made to email or telephone the 51 positive respondents to arrange an interview. We aimed to recruit 20–25 participants to achieve sufficient volume and richness of data from pragmatic sampling to enable a thematic analysis to be carried out.^9^

### Data collection

Semi-structured interviews were conducted by NT using a topic guide informed by the study aims and findings from the initial survey. Participants discussed their roles, changes to the delivery of community end-of-life care during the pandemic, opportunities for service innovation, and their concerns for the future delivery of end-of-life care during times of increased need. A copy of the topic guide is available as supplemental material.

Interviews were carried out on Google Meet between June and August 2021. Interviews lasted 27–52 minutes (mean = 42 minutes). Audio-recordings were transcribed verbatim, anonymised, and labelled with a unique study code. Data analysis was managed using NVivo v.12 and was undertaken consecutively, such that emerging themes could be explored in subsequent interviews.

### Data analysis

An inductive thematic analysis was conducted using Braun and Clarke’s process of data familiarisation, data coding, theme development and revision.^10^ Initial analysis and second coding of transcripts was undertaken separately by NT and AW to reduce the potential for lone researcher bias, with themes discussed and cross-checked for meaning and relevance through regular discussions with members of the research team (SM, CM, NT, AW).

Initial findings were presented to an online expert advisory group of general practitioners (n = 3), public health consultant (*n* = 1) and specialist palliative care consultants (*n* = 2) who had national leadership roles in palliative and end-of-life care (Royal College of General Practitioners/Marie Curie COVID-19 End-of-Life Care Thinktank). The discussion was recorded, reflected upon, and integrated with the study findings to develop recommendations for future practice in community end-of-life care.

### Ethical considerations

Ethical approval was granted by the University of Sheffield Research Ethics Committee [Ref: 035508]. Participants were provided with written information about the study and given the opportunity to ask questions during an initial telephone call. Given the potentially sensitive nature of the interview, they were made aware of their right to end the interview and withdraw from the study at any point, and information about support organisations was available. All participants returned a signed consent form by email prior to interview.

### Patient and public involvement

Patient and public involvement (PPI) was central to this research, with a PPI co-applicant joining the research team and co-authoring this manuscript. Further PPI was facilitated by the University of Sheffield Palliative Care Studies Advisory Group (PCSAG) prior to and during the study. Both our local PPI work and a national consultation exercise^11^ highlighted the importance of the provision of end-of-life care in the community during COVID-19. Group members provided comments on the initial project design and on the research questions and topic guide.

## Results

Interviews were conducted with 25 primary healthcare professionals: 8 general practitioners and 17 community nurses working within primary care. Participants were drawn from urban (*n* = 16), inner city (*n* = 4) and rural (*n* = 5) areas across the UK (Table 1). Table 1 describes the job role and location of the 25 study participants.

**Table 1.** Job role and location of the study participants (*n* = 25).

Job role	Description of Practice Area				Total
	Inner-city	Urban	Urban/Rural	Rural	
General practitioner	2	1	2	3	8
Community nurse	2	6	7	2	17
Total	4	7	9	5	25

Overall, three inter-related themes were identified from the data describing factors considered critical to providing palliative care in the community during the COVID-19 pandemic: partnership working, care planning and presence.

### Theme 1: Partnership working

Participants described the impact of both an increase in numbers of community patients needing end-of-life care during COVID-19, and greater complexity of individual care needs. Maximising the capability and opportunity for professionals to work together effectively was fundamental to addressing this. Delivering good community end-of-life care was described as ‘*a group achievement*’, referring to the need to work closely with other members of the primary care team and with specialist palliative care services. Ways of working that made a difference to fostering this approach included multi-disciplinary online staff education, training and peer support:

*Our local hospice did some [online] lunchtime sessions that were very supportive, and really most of our practice team clinicians attended those, whether they were at home or work, and I think we all learned a lot, but it also felt very supportive. I think it was good for people’s mental health as well, to feel that they had that sort of multidisciplinary support really, because a lot of people felt they were on very uncertain ground. [P14, inner-city general practitioner]*

Participants described virtual multi-disciplinary team meetings taking place online. Benefits included accessibility and opportunity for more professionals to join:

*Our MDT meetings are much easier now. We consistently can get the [specialist palliative care] nurse dialling in, and more of the district nurses dialling in, for example, and anyone else we need if it’s speech and language therapy because they’re a [motor neurone disease] patient or whatever, we can get them dialling in. So, I think technology has made it much easier for professionals to come together, and that benefits patients. [P15, rural general practitioner]*

For most participants, the ability to communicate online with colleagues from primary care teams and specialist services enabled and enhanced a sense of partnership working:

*I actually think that those relationships [with general practitioners and specialist palliative care services] have got even stronger because we’ve all had to play our part in it, we’re all cogs in a massive machine aren’t we, and we’ve all had a really important part to play. [P06, urban community nurse]*

A minority of participants felt the increased use of technology had resulted in a loss of personal connections, poorer relationships with colleagues and disengagement from collaborative working. The increased number of deaths that occurred at home drew attention to gaps in multi-disciplinary partnerships, especially with services aligned to healthcare, including social care:

*Probably the biggest thing that strikes me when people are in their own homes is access to the social care that they may require. I think in some cases it can be good, and emergency care packages can be put in place quite quickly, but sometimes, in my experience, I wonder if there’s a bigger role for helping families especially, who may be providing the bulk of care at home, to give them some extra support and respite and advice. [P11, urban general practitioner]*

### Theme 2: Care planning

Participants consistently described the importance of planning for changes in care needs from as early as possible in a person’s illness. Initiating earlier and more consistent conversations was associated with better advance and end-of-life care planning with patients. However, participants reported that time and resource for meaningful care planning conversations in primary care were lacking even before COVID-19. During the pandemic, there was a prevailing sense that conversations were having to take place in a hurry, during a crisis, and often remotely. These far from ideal experiences of communication with patients were often a source of practitioner distress. Some participants suggested that there would be benefits in a broad campaign to encourage the public to talk about their future care needs:

*I would love there to be a big campaign nationally about end-of-life care and about making these decisions, so advance care planning. I think it should be much more of a normal process that patients expect to be asked, whether or not it’s when they hit 75 or 80 or just a standard process, because at the moment I find people are genuinely horrified when you mention it. [P24, rural general practitioner]*

Some participants reported that they had increased their skills and confidence in initiating care planning conversations due to the COVID-19 pandemic:

*A good thing that’s come out of the pandemic, which I hope is here to stay, is that I guess we’re more open to the fact of having those [care planning] conversations and asking people where they want to be cared for. And I think that’s why we’re still seeing this influx of people dying at home, which is emotionally upsetting for us but then I guess at the same time, because we’ve got more confidence to have those [care planning] conversations, we can have them more freely, and more people are getting to die at home where they want to be. [P22, urban community nurse]*

There were some positive examples of increased time and resource allocation to care planning during the pandemic, including at times when patients were perceived to be ‘*self-managing a lot of things, or sitting on things’* and usual contractual arrangements for general practice were paused. One general practitioner described initiating projects with general practice registrars to improve care planning:

*We’ve done a huge amount of training, particularly on advance care planning and having good care planning discussions, because part of that is key to good palliative care. And we’re trying to work on enabling people to begin those conversations a lot earlier, and just offer people some information, maybe at more routine frailty reviews, that kind of thing. . . We’ve done a piece of work, where we’ve taken our housebound register, and cross referenced that with a severely frail register. So, we’ve picked up all the severely frail housebound, and the trainee [general practitioners], we’ve had almost a day a week to spend offering those very frail patients a sort of holistic review and introducing the idea of advance care planning. [P14, inner-city general practitioner]*

In terms of planning specific aspects of end-of-life care, general practitioners and community nurses reported having to ‘*do things differently*’ during the pandemic to ensure timely access to medications and equipment for symptom management. This included expanding electronic prescribing and creating ‘grab bags’ of medication that were easily accessible for community nurses, particularly out of hours:

*We probably worked a lot closer with the palliative care team. They put in place some grab bags, some end-of-life medication grab bags that were used at the hospital. So, if we went overnight and people didn’t have drugs, we’d come back and get the grab bag and take it out. And they also developed an online prescription, so [general practitioners] could do the end-of-life community prescription and do it electronically. [P10, urban community nurse]*

Long-standing concerns about inadequate electronic patient record sharing were further exposed during the pandemic. However, solutions that could be implemented quickly were limited:

*You can have really important detailed discussions in a really skilful way with someone, but then if you can’t share that so that the person who sees them at three in the morning has got access to that information, it’s almost just a wasted effort. [P15, rural general practitioner]*

### Theme 3: Physical presence

The third key theme related to the importance of the physical presence of general practitioners in the planning and delivery of end-of-life care. Virtually all the nurses interviewed expressed concerns that some general practitioners were not providing home visits. Community nursing teams therefore provided most hands-on end-of-life care. Where this happened, community nurses described feeling abandoned and unsupported:

*I felt that nurses were continually stepping up and filling that gap and it was all very much, [general practitioners] were ‘We can’t possibly put ourselves at risk’. Obviously, we went into loads and loads of care homes that were inundated with end-of-life patients. And in the end a lot of the senior nurses and nurse practitioners went in and did all the end-of-life prescribing, because a lot of the [general practitioners] didn’t. [P10, urban community nurse]*

The physical presence of general practitioners was considered important to maintaining partnership working in end-of-life care, including multi-disciplinary assessment of symptoms:

*Lack of face-to-face contact has been the biggest issue that’s caused all these other dilemmas . . . symptom control problems as well. . . Patients have basically had a lot more problems with symptom management. But it’s not our problem it’s their [patient’s] problem, because they are the ones who are suffering, aren’t they? [P04, urban community nurse]*

Some general practitioners implied they were adhering to policy guidance in moving to virtual consultations. Many of these participants expressed a strong sense of moral and emotional distress resulting from guidance to reduce face-to-face contact with patients dying at home. Some reported continuing with home visits despite guidance:

*I could not do this [end-of-life care] without being there, I couldn’t, it felt wrong. It needed the presence and, yes, I didn’t examine as carefully as I usually would have, I was scared, they were scared, but it needed me to go there. [P08, urban general practitioner]*

The presence of general practitioners in the home was key to maintaining the sense of community end-of-life care as a *‘group achievement’*, as described in the first theme. This included general practitioners working in out-of-hours services:

*We’ve been really pleased with the out-of-hours [general practitioners]. I think we have supported each other really well actually and that relationship has worked very well, they have come out quite a lot for us. [P04, urban community nurse]*

At a healthcare organisation level, the importance of the presence of primary care professionals ‘*at the strategic side of things’* to contribute to service planning, policy and practice guidance for end-of-life care was specifically highlighted:

*We need to tell commissioners, you must commission to support doctors and nurses, clinicians, to have good consultations with their patients, which include treating a presenting complaint, but would also include looking at the patient’s life, their context, and how they’re going to manage this in the future. [P09, urban general practitioner]*

Some participants suggested this would help to increase awareness of the central importance of primary care in end-of-life care, target resources to where they are needed and foster development of service delivery models in the community that are effective:

*It’s quite big policy changes really. What I don’t think we need is loads and loads more specialists in palliative care, but we’d like one or two more. But it’s quite big changes. It’s more about if the whole of our health and social care system worked differently these are the people that would benefit the most. [P15, rural general practitioner]*

## Discussion

### Main findings

The study findings provide insights into factors that affected delivery of community end-of-life care during the COVID-19 pandemic, a time of greatly increased demand. Partnership working was valued, with the shift to online meetings facilitating support and role-modelling from specialist palliative care. However, the physical presence of general practitioners was key to the effective partnership delivery of end-of-life care in the community. Online training and education was reported to enhance skills and confidence in the primary care workforce. Meaningful communication about advance and end-of-life care planning with patients was considered essential but depended on time and resource in primary care that was often in short supply, with staff struggling to create care planning opportunities and manage emotional demands.

### What this study adds

The delivery of end-of-life care in the community by primary care professionals was known to be under pressure before COVID-19. Barriers included a lack of skills and confidence amongst primary care practitioners, conflicting clinical and administrative demands on time, and poor communication between healthcare professionals involved in the patient’s care.^12,13^ Effective, collaborative working between primary care and specialist palliative care was inconsistent.^14^

This study provides understanding into the rapid changes in practice when demand for community end-of-life care escalated during the COVID-19 pandemic. Online multi-disciplinary team meetings between primary care and specialist palliative care were valued, as were collaborative online training and education opportunities. Enhanced training for the primary care workforce has consistently been identified as necessary for the development of community end-of-life care,^12^ although opportunities for multi-disciplinary learning were limited prior to the pandemic.^15^ In addition to enhancing skills, collaborative online learning can provide space for reflection, which may help to promote the well-being of practitioners during times of increased demand.^16^

Previous studies have called for an extension of palliative and end-of-life care training opportunities for general practitioners^17^ and community nurses.^18^ This study presents a case for developing and evaluating multi-disciplinary approaches to training and education, including the potential of digital technology. Building collaboration in training and in the development of policy and guidance has been identified as critical for the development of more flexible and resilient healthcare systems capable of responding to future increases in demand for end-of-life care, including in the community.^4^

## Developing recommendations for future practice

**Figure 1.**
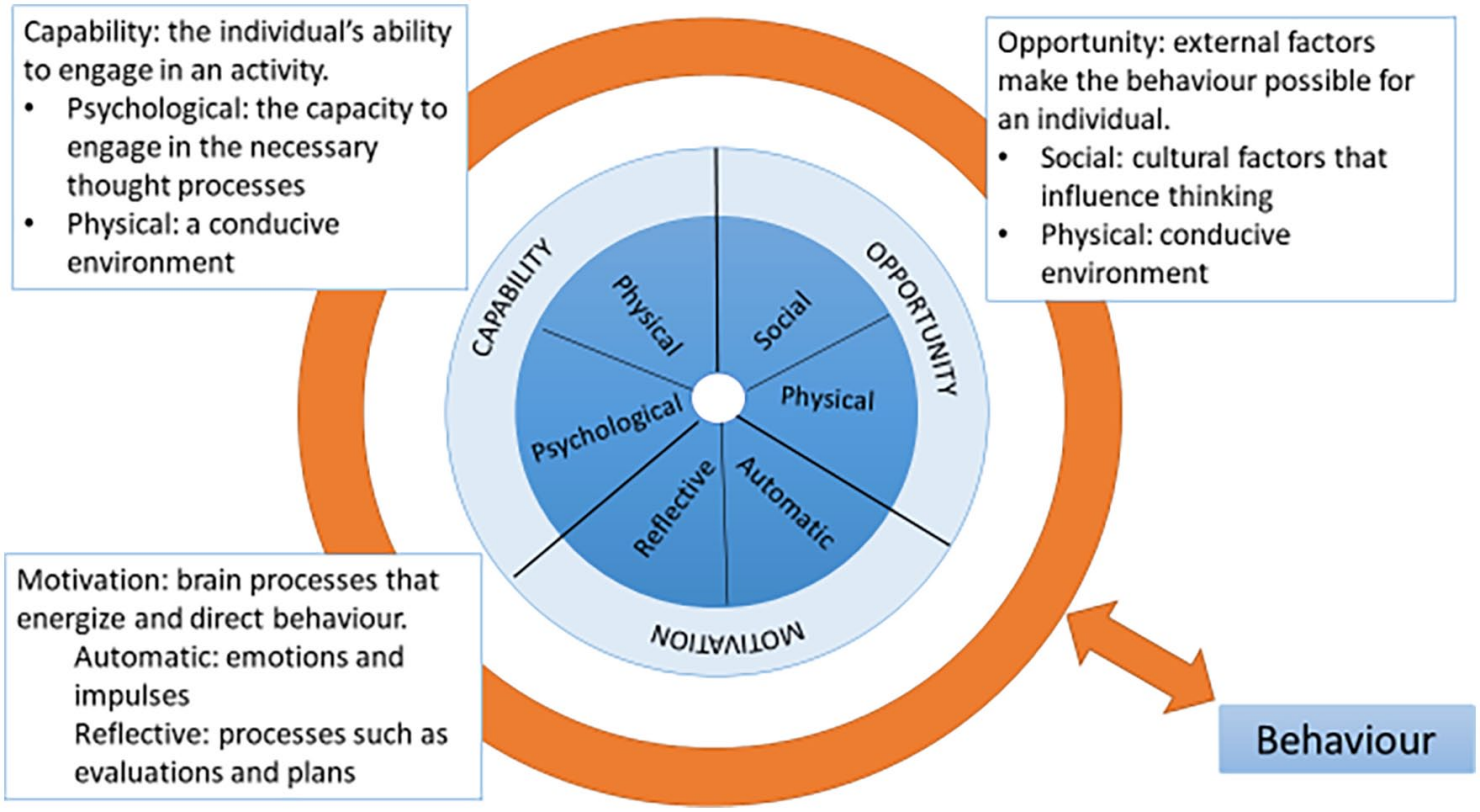
The COM-B behaviour system definitions, mapped across the Behaviour Change Wheel.

Behaviour change theory^19^ provides a framework through which to consider how interventions described as beneficial worked in practice during the pandemic in order to develop recommendations for future practice and policy. The Behaviour Change Wheel is underpinned by behaviour change theory, with capability, opportunity and motivation interacting to generate behaviour (the COM-B system). The Behaviour Change Wheel, and components of the COM-B system, are outlined in Figure 1:

Our findings suggest that enhancing the psychological capacity and automatic motivation (emotional response) of primary care team members, particularly community nurses, is an important consideration for future service design. Community nurses continued to deliver most face-to-face end-of-life care to patients at home but described a clear need for the presence of the general practitioner. It is likely that this related not only to the delivery of patient care, but also to the clinical leadership role of the general practitioner in a multi-disciplinary primary care team, taking responsibility for the management of clinical uncertainty and risk.^20^ Pandemic guidance issued separately to different professional groups in the primary care team led to tensions between team members and a lack of shared understanding that could be overcome by unified guidance in the future.

Another priority that emerged was the need for more time and resource for conversations with patients to plan for changes in health, including advance care planning.^14,21^ In addition, the need for effective systems to support the sharing of information on individual care preferences among healthcare providers, including primary care practitioners, that was identified reflects an enduring priority and an important area for research.^22,23^

**Figure 2.**
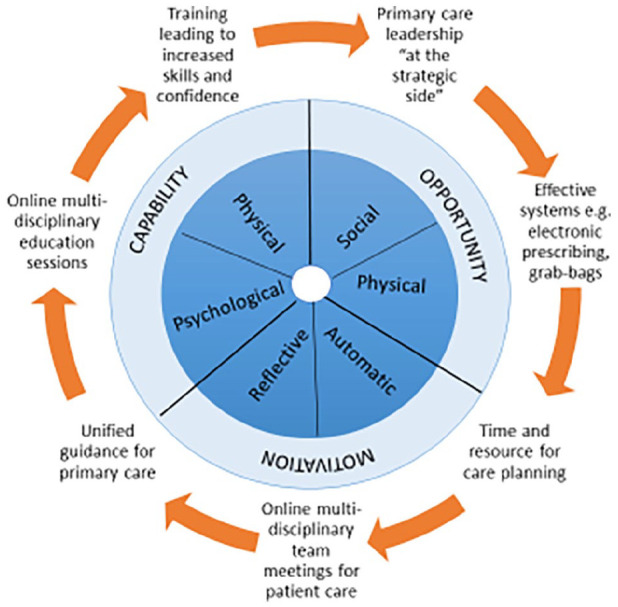
Beneficial interventions in COVID-19 community end-of-life care mapped to the Behaviour Change Wheel.

Following discussion with the expert advisory group, the implications for changes to practice in relation to community end-of-life care that emerged from the thematic analysis were mapped to the Behaviour Change Wheel (Figure 2) and used to inform the recommendations from the study. These are summarised in Table 2:

**Table 2.** Summary of recommendations.

Partnership working	Maintain multi-professional, cross-boundary clinical team meetings, with virtual meetings as an option.
	Continue to develop and deliver online collaborative training and education.
Care planning	Resource time and capability in primary care for effective communication about advance and end-of-life care planning with patients
	Maintain effective systems to support care planning, such as electronic prescribing. Provide clear, consistent and unified guidance for end-of-life care in primary care.
Physical presence	Recognise the importance of the physical presence of all members of the primary healthcare team in policy and practice guidance.
	Enhance and support effective primary care system leadership at local, regional and national levels.

### Strengths and limitations of the study

This study provides detailed, in-depth insights into the role and response of primary healthcare professionals in the delivery of end-of-life care in the community during the first two years of the COVID-19 pandemic (2020–21). It is one of very few studies to focus on this critical element of community end-of-life care delivery during a pandemic and builds on the rapid literature review and survey conducted by this research team.^3,6^

The study has limitations; participants were self-selecting, and their views may not be representative of the wider workforce. Participation was restricted to general practitioners and community nurses, and there is a need for more research into the experiences and perspective of other primary care team members including pharmacists, community therapists, paramedics, and general practice administrative staff. The study did not seek to include patient and family carer perspectives on the community end-of-life care they received. More patient-centred research is needed to increase understanding of the experiences and nature of services provided to people who died or continued to receive palliative and end-of-life care during the pandemic.

## Conclusion

Primary healthcare services have a key role in the delivery of community end-of-life care, yet there has been limited evidence to inform practice and policy when there is a rapid increase in demand for services, such as during a pandemic. This study has applied behaviour change theory to interpreting the unique perspectives of general practitioners and community nurses, and to considering how interventions developed during the COVID-19 pandemic enabled opportunity, enhanced capability and capitalised upon the motivation of the primary care workforce to provide community end-of-life care.

To embed positive change, an increased policy focus on primary care in palliative and end-of-life care is urgently needed. More collaborative research is required to consider factors that enable integration between primary care teams and specialist palliative care colleagues, with the aim of ensuring that good end-of-life care is a ‘group achievement’ available to all.

## Supplemental Material

sj-pdf-1-pmj-10.1177_02692163221140435 – Supplemental material for Role and response of primary healthcare services in community end-of-life care during COVID-19: Qualitative study and recommendations for primary palliative care delivery Click here for additional data file. Supplemental material, sj-pdf-1-pmj-10.1177_02692163221140435 for Role and response of primary healthcare services in community end-of-life care during COVID-19: Qualitative study and recommendations for primary palliative care delivery by Nicola Turner, Aysha Wahid, Phillip Oliver, Clare Gardiner, Helen Chapman, Dena Khan (PPI co-author), Kirsty Boyd, Jeremy Dale, Stephen Barclay, Catriona R Mayland and Sarah J Mitchell in Palliative Medicine
