# Supplementary material for: Role and response of primary healthcare services in community end-of-life care during COVID-19: Qualitative study and recommendations for primary palliative care delivery
Source: Palliat Med. 2023 Feb;37(2):235–43. doi: 10.1177/02692163221140435 (PMC9720421; doi:10.1177/02692163221140435)
Supplement: sj-pdf-1-pmj-10.1177_02692163221140435 – Supplemental material for Role and response of primary healthcare services in community end-of-life care during COVID-19: Qualitative study and recommendations for primary palliative care delivery [file sj-pdf-1-pmj-10.1177_02692163221140435.pdf]

PMJ-22-0169: Role and response of primary healthcare services in community end-of-life care during COVID-19: Qualitative study and recommendations for primary palliative care delivery.

Supplemental Material 1: Interview Topic Guide

**About you**<br>Please tell me about the work that you do / what do you do in your role?
**What are your roles in palliative and end of life care in the community?**<br>*Prompts:*<br>What is most important to you about this type of care?<br>What works? What doesn't work?
**How has your experience of palliative and end of life care in the community changed through the COVID-19 pandemic?**<br>*Prompts:*<br>In what ways has your practice changed? Personally / as a team
**Who else (any other services) do you work closely with?**<br>*Prompts:*<br>How could this be improved?<br>Is there anyone else who should be involved in your opinion?
**Where there any particular opportunities that arose as a result of the pandemic?**<br>*Prompts:*<br>Are there any innovations that you have seen happen that are particularly good?<br>What would you like to change (or not) in this area of practice in the future?<br>What would you tell service managers / commissioners was needed / needs to stay?
**Do you have any concerns about the future delivery of palliative and end of life care in the community?**
**Do you think that medical / nursing staff receive enough training in this area?**<br>*Prompts:*<br>Are there any specific issues that have arisen through the COVID-19 pandemic?<br>Have you had to learn new skills? What are they?<br>How have you learnt them?
**Can you describe a case that went well?**
**Can you describe a case where things didn't go well?**
